# Economic evaluation of health promotion for older people-methodological problems and challenges

**DOI:** 10.1186/s12913-016-1519-y

**Published:** 2016-09-05

**Authors:** Kai Huter, Ewa Kocot, Katarzyna Kissimova-Skarbek, Katarzyna Dubas-Jakóbczyk, Heinz Rothgang

**Affiliations:** 1 SOCIUM - Research Center on Inequality and Social Policy, University of Bremen, Mary-Somerville-Straße 5, 28359 Bremen, Germany; 2 High-profile area Health Sciences, University of Bremen, Bremen, Germany; 3 Health Economics and Social Security Department, Institute of Public Health, Jagiellonian University Medical College, Grzegórzecka 20 St., 30-351 Crakow, Poland

**Keywords:** Economic evaluation, Older people, Health promotion, QALY, Costs, Cost-effectiveness, Age-based-rationing

## Abstract

**Background:**

The support of health promotion activities for older people gains societal relevance in terms of enhancing the health and well-being of older people with a view to the efficient use of financial resources in the healthcare sector. Health economic evaluations have become an important instrument to support decision-making processes in many countries. Sound evidence on the cost-effectiveness of health promotion activities would encourage support for the implementation of health promotion activities for older people. This debate article discusses to what extent economic evaluation techniques are appropriate to support decision makers in the allocation of resources regarding health promotion activities for older people. We address the problem that the economic evaluation of these interventions is hampered by methodological obstacles that limit comparability, e.g. with economic evaluations of curative measures. Our central objective is to describe and discuss the specific problems and challenges entailed in the economic evaluation of health promotion activities especially for older people with regard to their usefulness for informing decision making processes.

**Discussion:**

Beyond general problems concerning the economic evaluation of health promotion, our discussion focusses on problems that pertain to the analysis of cost and outcomes of health promotion interventions for older people. With regard to costs these are general problems of economic evaluations, namely the actual implementation of a societal perspective, the appropriate measurement and valuation of informal caregiver time, the measurement and valuation of productivity costs and costs incurred in added years of life. The main problems concerning the identification and measurement of outcomes are related to the identification of outcome parameters that, firstly, adequately reflect the broad effects of health promotion interventions, especially social benefits that gain importance for older people, and secondly, ensure a comparability of effects across different age groups. In particular, the limitations of the widely used QALY for older people are discussed and recently developed alternatives are presented.

**Conclusions:**

The key conclusion of the article is that a comparison of the effects of different health promotion initiatives between different age groups by means of economic evaluation is not recommendable. Taking into account the complex outcomes of health promotion interventions it has to be accepted that the outcomes of these interventions will often not be comparable with clinical interventions and have to be assessed differently.

## Background

As a result of demographic change and a growing proportion of older people in the population, morbidity in this group has become a highly important issue, in particular with regard to increasing costs in the healthcare sector [1]. The support of health promotion activities for older people thus gains societal relevance in terms of enhancing the health and well-being of older people with a view to the efficient use of resources in the healthcare sector [2].

Establishing the best way to deploy limited societal resources and thereby maximise utility for the members of a given society is an issue of fundamental importance. The application of this principle in healthcare means deciding which share of the available resources should go into healthcare and its different branches and target groups. Bearing this in mind, this debate article discusses to what extent economic evaluation techniques are an appropriate means to support decision makers in the allocation of resources for health promotion activities for older people. To achieve this, the article examines different problems and methodological challenges of the economic evaluation of health promotion interventions in general – and discusses in detail problems and implications that are specific for the assessment of interventions aiming at older people. With a specific focus on health promotion for older people and on the usefulness of health economic evaluation to inform decision-making processes, this article thus adds to the broader literature on possibilities and limitations of health economic evaluation.

When political decisions about the funding of health promotion activities for special target groups need to be made, such activities have to be weighed up against measures for other target groups or curative measures which may be more effective or might have similar effects. *Economic evaluation* is defined by Drummond et al. as the comparative analysis of alternative courses of action in terms of both costs and consequences [3]. Applying this principle to healthcare provision, health economic evaluations have become an important instrument for supporting decision-making processes in many countries [4, 5]. Sound evidence on the cost-effectiveness of health promotion activities would encourage support for the implementation of health promotion activities for older people.

The basic tasks of any economic evaluation are to identify, measure, value and compare the *costs* and *consequences* of the alternatives under consideration. A full economic evaluation compares at least two alternatives and examines the costs and consequences of both of them. It applies the concept of opportunity costs, that is: benefits forgone when opting for a specific intervention [6]. The opportunity cost of a decision for a specific health promotion measure is the value of benefits of the next best alternative. Thus, economic evaluation helps to answer the question whether a specific programme is worth pursuing as against other programmes one could conduct with the same resources [3].

Originally developed for clinical interventions, health economic evaluation methods are today applied to more complex health promotion interventions and public health programmes. According to the WHO definition, “health promotion” is the process of enabling people to increase control over and improve their health. It moves beyond a focus on individual behaviour towards a wide range of social and environmental interventions [7]. Such strategies operate at multiple levels, including the individual, the family, the community, and society more generally. However, this broad focus tends to amplify general methodological problems of economic evaluations, especially the more complex and the less specific the targets of the respective programmes become. As a consequence, comparability with, for instance, health economic evaluations of curative measures may be limited.

Methodological problems pertaining to economic evaluations of health promotion activities in general have been widely discussed [e.g. 8–14]; a look at existing economic evaluations on health promotion, however, shows that for key problems only unsatisfactory solutions have been found. This is demonstrated for example in a review study by Weatherly et al. [11]. Target group-specific problems have not been discussed to a great extent so far in this context.

Nonetheless specific problems pertaining to economic evaluations of health promotion activities for older people are closely linked to problems concerning the economic evaluation of health promotion activities in general. With regard to cost-analysis the problems are linked to general unresolved issues in health economic evaluations that apply a societal perspective.

The aim of this paper is to map out the whole picture. For this reason we will outline in the following general problems of economic evaluations of health promotion activities. Based on this we will elaborate and discuss the specific problems and challenges that concern health promotion activities for older people. This particularly concerns problems of cost-analysis and the identification and measurement of appropriate outcomes, which will be discussed in more detail below. In relation to costing problems we draw on debates on general methodological challenges in health economic evaluations. This discussion implies not least the question whether the application of health economic evaluation methods might lead to age-based rationing and in particular to discrimination against health promotion and disease prevention for older people.

Finally, we will deduce some recommendations for the economic evaluation of health promotion activities for older people that particularly focus on their supportiveness for political decision-making processes.

## Discussion

Health promotion and preventive activities for older people comprise a wide range of activities, not all of which are complex long-term interventions. Programmes targeting individuals, such as fall-prevention programmes, pose less of a problem than population-based public health initiatives aiming more generally at promoting exercise and healthy nutrition, because a lot more influencing factors have to be considered. Methods for the evaluation of screening and immunisation interventions are well developed [11]. Nonetheless, there are additional target group-specific problems that have to be considered when evaluating health promotion or preventive interventions for older people. Table 1 provides an overview of the identified problems and their specific focus.

**Table 1:** Synoptic table of challenges concerning the economic evaluation of health promotion activities for older people

Synoptic table of problems concerning the economic evaluation of health promotion activities for older people
To differentiate the different aspects,
- general problems of health economic evaluations are in normal lettering;
- *aspects that arise mainly from the specific nature of health promotion are presented in italics*,
- **aspects that gain special importance for older people are in bold**
*Societal perspective recommended* (in some countries generally required)
Attribution of effects
- *Long time horizon* ***(but shorter effective period for older people)***
- *RCTs are difficult to implement*
- *influence of third variables is increased;*
- *Long term outcomes have to be estimated*
Measuring and valuing of outcomes
- *Multiple endpoints*
- *Long causal chains (use of proxy outcomes)*
- *Social benefits and non-health targets gain importance*
- *Intersectoral consequences*
- **Diverging preference structures of older people**
- **Cost-utility analysis and cost-benefit analysis may imply age based rationing**
Identification, measurement of costs
- **Measurement and valuation of informal caregiver time**
- Measurement and valuation of productivity costs **(including unpaid labour)**
- **Costs incurred in added years of life**
- *intersectoral costs*
*Equity considerations*
*Discounting of benefits reduces effectiveness results*
*Increased level of uncertainty*
*Choice of comparator is difficult*
*Context-sensitivity – limited transferability*

### General problems pertaining to economic evaluations of health promotion activities

Major difficulties relating to the economic evaluation of health promotion interventions compared to clinical interventions are related to the typically long time horizon and the broader objectives and possible effects of these interventions. These entail four key methodological challenges, namely the attribution of effects (1), the measurement and valuation of outcomes (2), the identification of intersectoral costs and consequences (3), and the incorporation of equity considerations (4) (as identified by Weatherly et al. [11]).

The problems concerning the *attribution of effects* are closely linked to the above-mentioned long *time horizon* of these interventions. This implies that the follow-up period of an evaluation study has to be correspondingly long – or modelling approaches have to be used. Interventions directed at populations are more difficult to evaluate by randomized controlled trials, which are the methodological gold standard when evaluating clinical interventions [15–17]. For this reason alternative study designs are often applied to capture the effects of an intervention.

The problems concerning the *measurement and valuation of outcomes* and those that pertain to the *analysis of costs* have specific implications for older people; these will be introduced and discussed in more detail in the next section.

The incorporation of *equity considerations* is of special interest because public health or health promotion interventions are often set up to counterbalance health inequalities. Instead of just maximizing health gains, they aim at an equity oriented distribution of health gains. Thus assessment criteria of health promotion interventions may differ significantly from clinical interventions.

Additional problems relate to the divergence of present costs and future benefits that are also linked to the long time horizon. To reflect the belief that, in general, society prefers to receive benefits sooner rather than later, the discounting of future benefits may reduce the effectiveness results of an intervention. In general the characterization of uncertainty by sensitivity analyses also has to be addressed [11, 18–22]. Last but not least since the political, organisational, social and environmental context of these programmes is often crucial [cf. 10, 14], the transferability of the results to other contexts may be limited.

After providing this general overview to problems concerning the economic evaluation of complex health promotion interventions, we will now turn to aspects that imply additional problems for older people as a target group for health promotion.

### Problems of identifying, measuring and valuating costs

Cost analysis, which implies the adequate identification of costs that have to be taken into account, their measurement and valuation, is one of the major components of the economic evaluation of health interventions. There are different types of costs to be considered in a cost analysis, namely: resources used (costs) in the healthcare sector, resources used in other sectors (and covered e.g. by municipal budgets), resources or costs incurred by the patient or programme participant and their relatives, and productivity losses [3]. At this point we want to highlight four aspects relating to costs that pose methodological challenges that are not easy to resolve. These are: the recommended cost perspective of the study (1), the appropriate measurement and valuation of informal caregiver time (2), the measurement and valuation of productivity costs (3) and the (healthcare) costs incurred in added years of life (4). While these aspects are critical issues in costing methodology of health interventions in general (as identified e.g. by Meltzer and Smith [23], [cf. 24]), their influence may be especially high in economic evaluations of health promotion in general and for interventions aiming at older people in particular.

### Perspective of the analysis

Health economic evaluations can be conducted from different perspectives, chiefly that of the patient or programme participant, the provider, the third-party payer or society as a whole. The perspective of the analysis determines which costs and consequences are to be included. For the economic evaluation of health promotion interventions the societal perspective is generally recommended [9, 10, 24], though in practice the analysis is often conducted from a provider or public-payer perspective (by national HTA-agencies) [11, 25]. Nonetheless, some national guidelines generally demand the use of the societal perspective (e.g. Netherlands and Sweden). The societal perspective captures the value of all changes in resources used and gained as a result of an intervention, including informal caregiving and productivity costs. As health promotion activities often affect different sectors, like education, health services, long-term care or environmental strategies, and rely on volunteer work or participants’ leisure activities, evaluation results will be biased if not all implied costs and consequences are identified, even if no actual payment is made for some of those resources. This affects especially complex, multi-level or multi-strategy approaches. With respect to older people this may especially concern costs related to long-term care and unpaid work such as informal care or volunteer work. Adopting a single agency perspective is likely to result in a partial evaluation that may exclude major costs and benefits simply because they fall on different sectors. To fully account for all costs and benefits of a health promotion intervention, intersectoral impacts have to be quantified as far as possible. If this is not possible, they should be described at least qualitatively.

### The appropriate measurement and valuation of informal caregiver time

Caregiving costs – needed or saved due to an intervention – have to be included in the cost analysis, though often only paid caregiving is considered [4]. If the value of informal, non-professional and non-compensated care – provided by family members, friends or neighbours – is disregarded, the true value of an intervention’s costs and benefits may be underestimated. In addition to several types of costs that are incurred to facilitate informal care and effects on the health or well-being of the caregiver, the time spent by a caregiver is the major element of informal care and difficult to value. A systematic review on methods used for and implications of including informal care in applied economic evaluation shows that to date only a small proportion of economic evaluation studies include informal care and that no consensus exists on how to incorporate informal care. The extent of the impact varies from study to study [26].

Correct measurement and valuation is crucial for a complete cost analysis of interventions for older people, who are usually both providers and receivers of informal non-paid care – especially as it can be a defined target of health promotion for older people to avoid dependency on long-term care. Different methods of measuring and valuing informal caregiving costs have been proposed, like the proxy good method, the opportunity cost method, the contingent valuation method (CVM), the conjoint measurement method (CM) and the well-being valuation method (WBM) [20, 27–31]. While the proxy good and the opportunity cost method value the time spent on informal care only, the CVM, CM and WBM are based on preferences and (may) include a valuation of effects on health and well-being as well and thus imply a certain risk of double-counting health-effects. A detailed discussion of the benefits and risks of these methods for different types of economic evaluation (depending on outcome measures) is provided by Koopmanschap et al. [32]. They show quite clearly that the way in which informal care is incorporated needs careful consideration. Ignoring these cost aspects may bias economic evaluations of interventions for older people considerably. As older people may receive *and* provide informal care the time invested in informal care is considered in costing analysis as (i) direct non-medical cost, which may be reduced as a result of intervention and (ii) productivity cost, i.e. value of time for caregiving by the participant which is gained as a result of intervention.

### The measurement and valuation of productivity costs

Productivity costs primarily represent lost economic productivity due to death (mortality costs), or lost or impaired ability to work or to engage in leisure activities due to morbidity (morbidity costs) [3]. Productivity benefits or losses can accrue to the individual in terms of individual income, workforce activity, informal caregiving, and leisure activities, or to the family in the form of reduced caretaking costs and reduced impact on household income. Whether and how productivity costs should be included in health economic evaluations is widely debated, both in theory and practice [cf. 20, 33–39].

Due to a lack of consensus on the inclusion of productivity costs and the absence of a standard method for estimating them, productivity costs are often disregarded in actual economic evaluations, as testified in an outstanding review paper by Krol et al. [39]. Whether productivity costs should be included or not is ultimately a normative question and depends on the normative framework of the evaluation. A welfarist perspective will include productivity costs; an extra-welfarist perspective will not weight health gains against productivity costs.

There are three main approaches for measuring and valuing productivity costs, and they were discussed extensively in the early 1990s: the human capital, the friction cost and the willingness to pay approach (WTP). The main criticism of the human capital approach is that it values life time in terms of individual earnings. Thus, the human capital approach may economically undervalue the productivity costs of older people, who are engaged predominantly in unpaid work. Although the WTP design can address the limitations of the human capital approach, it has been more difficult and costly to implement and has been used in relatively few cost studies. The concept behind the friction costs approach is that production losses due to illness may not be as great as expected, because existing labour pools and workplace structures can absorb some of this lost productivity. Unlike lost time from paid work that is measured and valued here mainly by the friction period, the replacement of older persons’ informal caregiving (to a beloved person or close relative) is often not feasible. In such cases the costs caused by the reduction of their productivity will be underestimated. The choice of which method to use in a study will significantly influence the overall results. Estimates based on the willingness to pay approach, for example, are generally considerably larger than those generated by a human capital approach. The friction cost approach usually results in the lowest cost of the three designs [3, 36, 37]. Beyond that, there is a debate whether productivity consequences are included in quality-of-life outcomes of an intervention, so double-counting has to be taken into account [e.g. 37].

If productivity costs are included, the analysis is mostly focused on productivity related to paid work [39]. This focus neglects the value of the contribution to society provided by unpaid work e.g. of seniors, like volunteer work, household work or informal care for relatives, neighbours or children. Thus, the inclusion of productivity costs in the economic evaluation will discriminate against seniors if unpaid work is not included or valued notably less than paid work. How this valuation should be done has to be subjected to further scientific debate, for more references to this debate see e.g. Krol et al. [40] (see also references on informal caregiving).

### Costs incurred in added years of life

There is a broad debate over whether costs that incur in life years gained by an intervention should be included in a health economic evaluation or not [41]. While there is consensus that changes in future medical and non-medical costs *related to* the intervention should be included in the cost analysis, less agreement exists over unrelated costs. In guidelines on how to conduct economic evaluations the choice whether or not to include *unrelated* medical costs in life years gained is mostly left to the analysts, or these costs are explicitly excluded [27, 33, 41]. In the more recent theoretical debate, arguments in favour of including unrelated healthcare costs are gaining support [25, 41]. Meltzer argues that interventions that enhance life quality will be disadvantaged over interventions that prolong life if these costs are not considered [42, 43]. A more general argument is that if health gains in terms of life years gained are included in the numerator of the cost-effectiveness ratio, respective costs have to be included for the sake of consistency [3].

As a majority of healthcare costs accrue in the last year of life [44] and costs in the last year of life are generally lower for older than for younger people, costs of dying could decrease by delaying mortality [45]. Consequently a life-prolonging intervention could lead to a reduction of future medical costs [46]. This might gain even further relevance if future healthcare costs are discounted to present value. On the other hand, an extended lifespan increases the probability of the occurrence of additional, maybe costly diseases [47].

The inclusion of costs unrelated to a health promotion intervention for older people may imply that life-prolonging interventions will be rated less cost-effective for older people, because the present value of these costs will be higher due to its appearance in near future.

Another problem of unrelated costs in life years gained lies in the practical issue of how to find reliable estimations, or how to include these costs in general, while taking into account that healthcare expenditure depends notably on proximity to death rather than age in particular [41, 48]. For this reason it is generally recommended to perform sensitivity analyses to assess the impact of unrelated costs in added life years on the cost-effectiveness ratio; in this way, the impact of including or excluding these costs will be demonstrated. Additionally it has to be assessed whether sufficient reliable data on future costs for the specific population exist.

This short overview of important aspects of cost analysis in a societal perspective illustrates that in the economic evaluation of health promotion interventions for older people special attention has to be paid to these aspects in order to avoid biased results to the disadvantage of older people. These limitations have to be assessed carefully to assure comparability.

### Problems in health promotion outcome evaluations

Outcome evaluations comprise three main steps, regardless of the subject of the analysis (medical interventions, health promotion programmes, drug therapies etc.). These are identification, measurement, and valuation. In the case of health promotion initiatives for older people, specific problems occur at each of these steps.

### Identification of outcomes

The identification of outcomes is not the primary task of health economic evaluations. Health economic evaluations are usually based on the analysis of outcome measurements originally carried out e.g. by physicians who identify effects according to the standards of evidence-based medicine (EBM) [25]. The proof of the efficacy and effectiveness of an intervention rather has to precede the health economic evaluation [3]. Specific requirements of health economic evaluations consist, firstly, in condensing the effects of an intervention to an outcome parameter that can be compared with the costs and, secondly, in the consideration of a time frame that will usually exceed the observation period of a randomized controlled trial (RCT) [25].

The ultimate effects of health promotion activities comprise not only health (mortality, morbidity), but also social outcomes (quality of life, functional independence, equity). Social outcomes may only be noticeable after a considerable length of time. To make up for this, changes more closely connected with health promotion programmes are considered as *proxy outcome indicators*. These can be immediate results of an intervention, or so-called intermediate health outcomes like improved health literacy, healthy lifestyles, social activities that affect health determinants or the enhancement of healthy organisational practices (cf. Nutbeam’s categorization [12]). Proxy outcomes, however, have similar problems to “surrogate outcomes” in clinical studies; they are substitutes for patient-relevant endpoints, but an actual causal relationship may be difficult to prove. Clinical studies provide many examples for assumed surrogate markers (e.g. reduced blood pressure) that do not ultimately affect patient-relevant outcomes in terms of mortality, morbidity or quality of life. Proxy outcomes need to be validated if they are used in economic evaluations. Because effects of health promotion interventions unfold over a long period of time, they may be influenced by other parameters, programmes or contextual conditions. To prove in detail the individual steps of a correspondingly long causal chain is a challenging task for the impact evaluation of health promotion activities. Proxy outcomes thus have to be used with great caution.

Health economic evaluations usually consider changes in the state of health or in personal benefits as relevant outcomes, according to EBM standard relevant effects have to be proven for individuals. This approach is suitable for preventive measures, but health promotion activities aiming at non-health targets like empowerment, social inclusion or the reduction of social inequalities will be disadvantaged here, because these social benefits are difficult to account for. Health promotion activities may have significant externalities, because the impact of a programme may affect not only the participating individuals, but also their families, friends and communities; these are mostly assumed to be positive, but negative side effects may be underestimated as well. Implications of these so-called spillover effects in cost-effectiveness analysis have been discussed and conceptualized for example by Basu and Meltzer [49]. Equity considerations in particular are an important issue in health promotion and have to be taken into account [11, 50]. But targets like these can only become relevant in health economic evaluations if they are part of the individuals’ benefit assessment (welfarist notion) or if they are explicitly and additionally accounted for (extra-welfarist notion) [25].

A focus on health impacts only is particularly problematic in the context of health promotion interventions for older people. For older people it is often difficult to distinguish between health and social needs, thus the social value of a programme may be more important than health improvement. Integration into the community, inclusion, or increasing mobility are not always associated with an improved health status, but they can be crucial outcomes of a health promotion programme. Therefore, taking the “beyond health” benefits into account is particularly important in the case of programmes for older people.

### Measurement and valuation

The measurement of health promotion outcomes is difficult in the light of the complexity and the multidimensionality of effects. In health economic evaluation three different types of outcome parameters are distinguished: *natural parameters* that are used in cost-effectiveness analysis (CEA), *virtual parameters* used in cost-utility analysis (CUA), and *monetary outcomes* as part of a cost-benefit analysis (CBA).

#### Problems concerning CEA

*Natural parameters,* for example weight loss, new cases of disease that were prevented (heart attack, stroke) or life years gained, measure only one-dimensional effects. Their comparability is mostly limited to indication-specific interventions and they appear to be unsuitable for covering multidimensional or complex effects of health promotion. The juxtaposition of several natural outcome parameters also reduces the comparability of different interventions.

As discussed above, proxy or surrogate parameters that are sometimes used where direct health status improvements are difficult to identify have to be used with great caution [51]. The application possibilities of CEA are thus very limited. It is possible to evaluate preventive measures with narrowly defined objectives, but the disadvantage here is that in the majority of cases results cannot be compared across different indicators.

#### Problems concerning CUA

A common concept for covering the multidimensionality of effects and achieving a comparability of different types of interventions is the use of a comprehensive index – a virtual parameter – that identifies, quantifies, evaluates and sums up a variety of effects. A prominent concept for measuring effects in healthcare is a quality of life (QoL) index. The most commonly used indicator is the quality adjusted life year (QALY). It takes into account both quantitative (life extension) and qualitative (health-related quality of life) effects of an intervention. The QALY serves as a widely accepted reference standard for health economic evaluations and is also commonly used in the evaluation of health promotion interventions. Although the QALY offers these distinct advantages, it entails several limitations and methodological problems that have been widely discussed [e.g. 52–55]. In conceptual debates it is criticised that QALYs derive from an extra-welfarist perspective. The utility to be maximised is determined externally by policy-makers. The relevant dimensions (quality and time), and their linear conjunction have been set externally; they do not derive from the individuals’ preferences. Technical problems relate especially to the fact that different instruments to value QoL yield different results.

Especially when it comes to the evaluation of health promotion interventions for older people, the possible uses of QALYs are very limited. There are four main reasons why the use of QALY may discriminate against older age groups [4], [cf. 56–59]. First, as older people – even if they are healthy – have a lower remaining life expectancy, the possible gain in (quality-adjusted) life years is lower compared to that of younger people. As a result, the number of QALYs that might be “produced” by an intervention is smaller and in consequence the intervention will be less highly valued. Second, instruments that assess QoL are usually limited to health-related quality of life (e.g. EQ-5D, SF-36), so individual beyond-health effects like maintaining independence or the social effects of an intervention – which, as already explained above, are particularly important for older people, will not be captured. Third, instruments that assess QoL measure the quality of life regardless of age. In common QoL indexes like EQ-5D, physical functionalities are of particular significance, though these become less important with increasing age. The QoL of older people will be underrated if age-dependent measures of value are not considered. Fourth, the average health condition of older people is poorer than that of younger people. Due to co-morbidities the health gains that can be realised are smaller, since even if an intervention is successful it will not restore full health. At the same time, small health gains are measured rather poorly by instruments used to assess QALYs. This is a reason why health promotion interventions for older people can lead to fewer measured health benefits, and thus to a comparably less improved quality of life, even though the objectives of an intervention have been fully achieved.

Given these limitations, the QALY is not very suitable as an outcome measure for older people. In particular with respect to the preferences of different age groups the underlying, oft-cited notion that “a QALY is a QALY is a QALY” [60], has to be rejected. If it is taken into account that preferences and especially health-related preferences change during the life course, quality of life should not be measured by a universal indicator. On the other hand, this implies that a comparison of QoL indexes between different age groups is not recommendable, and that comparability is limited to different interventions within the age group of older people. Preference-based approaches are in principle suitable for measuring multidimensional effects on an individual level, but they have to take specific preferences of the target group into account, and have to include social dimensions as well if they are to cover the effects of health promotion interventions for older people adequately. Different thresholds for different age groups are not a satisfactory solution, because relevant QoL dimensions for older people are not covered by the indicator.

#### Recently developed alternatives to measure QoL of older people

In the light of the QALY’s shortcomings, other instruments have been proposed to measure QoL for older people which are not limited to health-related QoL but assess a broader scope of well-being. In a recent review of QoL instruments for economic evaluations in health and social care for older people, Makai et al. identified four promising well-being instruments: the Ferrans and Powers QLI, the WHO-QoL-Old, ICECAP-O and the ASCOT. The first two instruments are widely validated, but lack preference weights. On the other hand, preference weights are available for ICECAP-O and the ASCOT, but they are less widely validated [61].

The *ICECAP-O* (ICEpop CAPability measure for Older people) – as one example – is a recently developed index to assess quality of life or well-being beyond health especially for older people, and is conceptually linked to Sen’s capability approach [62, 63]. It has been developed on the basis of an extensive qualitative survey about important determinants of quality of life among older adults in the UK. The focus of the measure is on capabilities (what people are able to do) rather than on functioning (what people do). It consists of five attributes: attachment (love and friendship), security (thinking about the future without concern), role (doing things that make you feel valued), enjoyment (enjoyment and pleasure) and control (independence). It is currently being validated and tested in different settings, [e.g. 64–68].

By reflecting actual value orientations of older people and by including social aspects of well-being, the ICECAP-O and the ASCOT are promising instruments for measuring quality of life of older people and in particular target-dimensions of public health interventions. However, the comparability of results is limited to different interventions within the older age group. Since they might also be limited in capturing health dimensions, Makai et al. recommend the use of health measures like the EQ-5D alongside the ICECAP-O or ASCOT to explicitly capture health benefits as well [61].

#### Problems concerning CBA

Another option for overcoming the limitations of natural or QoL parameters is the *monetary valuation of effects* (CBA). This can be done without subjective elements by determining the health costs avoided. Since this would imply that the health gain itself is attributed no value, another means of monetisation is used more commonly by evaluating the effects of an intervention through the individual willingness to pay for it. The individual’s *willingness to pay* captures different effects of an intervention in one unit. It is up to the individual to balance and weight the different values or benefits, and non-health related effects can be included as well. This allows a comparison of measures in different policy fields. It corresponds to a welfarist normative position [25].

A major problem of willingness to pay approaches in this context is the dependency of the result on the respondent. If measures are clearly limited to a defined target group – like older people – the vote on an allocative question will be biased by the distributive consequences for the respondents. Assuming divergent value orientations of different age groups, willingness to pay for a preventive measure for older people will not be independent of whether younger or older people are asked. Other difficulties in the application of CBA on health promotion interventions relate to the fact that these interventions sometimes have features of public goods and people report very low willingness to pay for them, if at all. Moreover, willingness to pay is dependent on an individual’s ability to pay.

Though not precisely a discrete method, some authors recommend the application of *cost-consequence analyses* (CCA) especially in the field of health promotion or public health interventions [11, 69]. Characteristic for a CCA is that the different cost components and the various outcomes of an intervention are presented and calculated separately. It takes into account the fact that there are different types of benefits that might be difficult to aggregate or might be assessed differently from various perspectives. It can be described as a CEA or CUA with multiple outcomes. CCA allows decision makers to decide on the basis of a differentiated assessment of the various effects or benefits and this allows them to set specific priorities. Still, comparability with other interventions is limited.

A systematic overview of the pros and cons of the different outcome indicators referring to different types of economic evaluation is presented in Table 2. As the choice of appropriate outcome indicators is of particular importance for the economic evaluations of health promotion, we put a special focus on this.

**Table 2:** Overview on pros and cons of different outcome indicators in economic evaluations of health promotion for older people

Outcome Indicators	Cons	Pros
Cost effectiveness analysis (CEA)		
Natural indicators	- Effects are reduced to a single parameter - Not all effects are covered (e.g. intersectoral effects) - Relevant health promotion outcomes are often difficult to operationalize - Often proxy outcomes are used (causality to patient-relevant endpoints has to be proven) - Comparability of different interventions is very limited	- Depending on the operationalization mostly easy and clearly measurable - Disease specific comparisons are very easily possible - Even small health gains can be documented - Simple and straightforward comparability of the one given indicator
Cost utility analysis (CUA)		
Aggregated indicators in general	- Aggregation of different aspects will always represent a limited perspective	- Multidimensionality of effects can be covered - Different types of interventions can be compared
e.g. QALY	- Only health related - Social benefits are not covered - Intersectoral benefits are not covered - The linear conjunction of time and quality is set externally (not preference based) - Limited comparability for HPA - Small health gains are measured poorly - Does not reflect preferences of older people appropriately - May discriminate against older people	- Widely accepted reference standard and well established instrument that is used in many economic evaluations
e.g. ICE-CAP-O	- So far not widely validated - No comparison across age groups possible - Limited in capturing health dimensions	- Developed according to preference weights of older people
Cost benefit analysis (CBA)		
Measurement of health costs avoided only	- Health gains or social benefits are attributed no value	- Subjective elements are excluded - Allows the comparison of measures in different fields of policy
Monetary valuation of outcomes e.g. by willingness to pay	- Political reservations against monetary valuation of health benefits - HPA have features of public goods, resulting in low willingness-to-pay - Results are not independent of who is asked: thus an age bias is possible - Willingness to pay is dependent on ability to pay	- Allows the comparison of measures in different fields of policy - Non-health benefits can be included
Cost consequence analysis (CCA)		
No standalone method CEA or CUA with multiple endpoints	- Limited comparability of different interventions - Complex results may be difficult to interpret	- Very transparent - Intersectoral costs and benefits can be covered - Contentious cost categories can be included - Broadly spread effects are at least described qualitatively - Description of equity effects can be included - Enables decision makers to decide based on a differentiated assessment of the various effects or benefits - Allows the setting of specific priorities

## Conclusions

The starting point of this article was the question to what extent economic evaluation techniques are an appropriate means to support decision makers in the allocation of resources for health promotion activities for older people, as the evaluation of health promotion activities for older people entails specific problems, which have to be taken into account. Disregarding these problems could implicitly lead to discrimination against health promotion measures for older people and thus an age-based rationing of public healthcare. Key problems concerning the economic evaluation of health promotion activities in general and problems with a special impact on older people are summarised in Table 1.

For each evaluation choices on perspective, outcomes and costs have to be made, which influence the result. Table 3 summarizes how results are influenced based on certain decisions.

**Table 3:** Risk factors for age discrimination in the economic evaluation of health promotion for older people

Methodological options	Potential discriminatory effects for older people
**If …**	**the effect will be ….**
the perspective of the study is partial,	societal benefits are underestimated; for older people e.g. reduced costs for long-term care.
informal caregivers time and other informal care costs are excluded,	benefits of interventions that aim at the reduction of dependency on long-term care are underestimated.
productivity costs are included without considering unpaid work,	societal value of senior’s unpaid work is neglected (informal care, volunteer work, household work).
cost incurred in added years of life unrelated to the interventions are included,	life-prolonging interventions for older people will be rated less cost effective, because older people will produce more costs in near future due to comorbidities.
effects are measured by natural parameters (CEA),	social benefits that are more important for older people are not covered.
effects are measured by QALYs (CUA),	benefits of interventions for older people will be underestimated, because
	… preferences of older people, especially social benefits are not covered.
	… a lower life expectancy results in less QALYs gained.
benefits are valued as monetary outcomes by willingness-to-pay (CBA),	results will be biased depending on distributive effects on the respondent, interventions for older people may be rated poorly if respondents are younger people.
benefits are valued monetarily without subjective elements (CBA),	benefits of the intervention will be underestimated, because social benefits are especially important for older people.

Based on the argumentation put forward in this paper regarding these problems, the main conclusions can be summarised as follows.

A key conclusion of this paper is that a comparison of the effects of different health promotion initiatives between different age groups by means of economic evaluation is not recommendable.

Due to the greater complexity of the effects, health promotion and curative interventions will seldom be directly comparable with clinical interventions. A major problem for the economic evaluation of health promotion activities thus consists in the identification or definition of *appropriate outcome indicators* that are directly connected with the formulated target of the intervention and ensure the comparability of different measures.

Aggregated indicators – like the QALY – that focus on health-related effects only are particularly inappropriate for covering the preference structures of older people. The time component constitutes an additional risk of discrimination against interventions for older people. If QALYs are used, they have to be used with great caution, and comparisons have to be limited to comparisons within a specific age group. More research on adequate age-specific preference-based outcome indicators such as the ICECAP-O is necessary.

Proxy outcomes should be used with caution, and even then only proxy outcomes whose effect on patient-relevant outcomes is thoroughly proven. The monetary valuation of effects by willingness to pay allows the inclusion of intersectoral effects of health promotion, but implies the risk of age discrimination as outcomes depend on the age of the respondent.

Taking into account the complex outcomes of health promotion interventions it has to be accepted that not all effects can be combined into a single index. Social benefits, equity considerations, sociocultural effects can rather be described qualitatively.

As a comparison of interventions for different age groups is not recommendable, the resource allocation between health promotion interventions for different age groups is ultimately a social or political decision that has to be made at the political level. These allocation or distribution decisions cannot be deduced directly from economic evaluations. The application of appropriate instruments will allow a comparison of the effects of interventions within specific age-groups. Given the quantity of unresolved methodological problems relating to costs, the consideration of contentious cost-categories implies a high degree of uncertainty. As the impact on interventions for older populations may be substantial, the results of their economic evaluation should be presented in a sensitivity analysis with and without these cost categories. The disclosure of contentious costs in a cost-consequence analysis will allow policymakers to decide whether they will be relevant for the decision.

If decisions over the allocation of resources between health promotion and clinical interventions depend on the availability of economic evaluations or cost-effectiveness studies, economic evaluations of health promotion activities have to be promoted. They are feasible, but they are considerably more complex than clinical evaluations – and they have to be assessed differently.

The more complex and multidimensional a health promotion activity is, the more difficult it will be to obtain evidence on an economic evaluation that covers all possible costs and effects; this concerns interventions addressing older people all the more as the economic evaluation entails additional problems. This implies the risk that – if the funding of health promotion activities is made conditional on the evidence of cost-effectiveness – complex measures are less likely to be funded.

## Abbreviations

CBA, cost-benefit analysis; CCA, cost-consequence analysis; CEA, cost-effectiveness analysis; CUA, cost-utility analysis; EBM, evidence-based medicine; QALY, quality-adjusted life year; QoL, quality of life

## References

[1] Westerhout E (2014). Population ageing and health care expenditure growth. International Handbook on Ageing and Public Policy.

[2] Mathar T, Jansen YJFM. Health Promotion and Prevention Programmes in Practice: How Patients’ Health Practices Are Rationalised, Reconceptualised and Reorganised. Bielefeld: transcript; 2010.

[3] Drummond MF, Sculpher MJ, Torrance GW, O’Brian BJ, Stoddart GL (2005). Methods for the Economic Evaluation of Health Care Programmes.

[4] Edlin R, Harper S, Hamblin K (2014). Assessing the cost-effectiveness of therapies for older people. International Handbook on Ageing and Public Policy.

[5] McDaid D, Sassi F, Merkur S, McDaid D, Sassi F, Merkur S (2015). Supporting effective and efficient policies: the role of economic analysis. Promoting Health, Preventing Disease. The economic case.

[6] Palmer S, Raftery J (1999). Opportunity cost. BMJ.

[7] World Health Organization (1986). Ottawa Charter for Health Promotion.

[8] Cohen D (1994). Health promotion and cost-effectiveness. Health Promot Int.

[9] Edwards RT, Charles JM, Lloyd-Williams H (2013). Public health economics: a systematic review of guidance for the economic evaluation of public health interventions and discussion of key methodological issues. BMC Public Health.

[10] Hale J (2000). What contribution can health economics make to health promotion?. Health Promot Int.

[11] Weatherly H, Drummond M, Claxton K, Cookson R, Ferguson B, Godfrey C, Rice N, Sculpher M, Sowden A (2009). Methods for assessing the cost-effectiveness of public health interventions: Key challenges and recommendations. Health Policy.

[12] Nutbeam D (1998). Evaluating health promotion—progress, problems and solutions. Health Promot Int.

[13] Richardson J (1998). Economic evaluation of health promotion: friend or foe?. Aust N Z J Public Health.

[14] de Salazar L, Jackson S, Shiell A, Rice M (2007). Guide to Economic Evaluation in Health Promotion.

[15] University of York, NHS Centre for Reviews and Dissemination (2009). Systematic Reviews: CRD’s Guidance for Undertaking Reviews in Health Care.

[16] Rychetnik L, Frommer M (2002). A Schema for Evaluating Evidence on Public Health Interventions.

[17] Centers for Disease Control and Prevention. Introduction to Program Evaluation for Public Health Programs: A Self-Study Guide. U.S. Department of Health and Human Services. Centers for Disease Control and Prevention. Office of the Director, Office of Strategy and Innovation, Atlanta; 2011.

[18] Cairns J, Drummond M, McGuire A (2001). Discounting in economic evaluation. Economic Evaluation in Health Care: Merging Theory with Practice.

[19] Claxton K, Paulden M, Gravelle H, Brouwer W, Culyer AJ (2011). Discounting and decision making in the economic evaluation of health-care technologies. Health Econ.

[20] Gold MR, Siegel JE, Russell LB, Weinstein MC (1996). Cost-Effectiveness in Health and Medicine.

[21] Gravelle H, Brouwer W, Niessen L, Postma M, Rutten F (2007). Discounting in economic evaluations: stepping forward towards optimal decision rules. Health Econ.

[22] Lazaro A, Barberan R, Rubio E (2002). The economic evaluation of health programmes: why discount health consequences more than monetary consequences?. Appl Econ.

[23] Meltzer DO, Smith PC. Theoretical issues relevant to the economic evaluation of health technologies. In: Pauly MV, McGuire TG, Barros PLP, (eds). Handbook of Health Economics Vol. 2. Amsterdam [etc.]: North Holland; 2012. pp. 433–469.

[24] Mogyorosy Z, Smith P (2005). The Main Methodological Issues in Costing Health Care Services: A Literature Review, CHE Research Paper 7.

[25] Rothgang H, Salomon T, Kolip P, Müller V (2009). Die ökonomische Evaluation von Gesundheitsförderung und Prävention. Qualität von Gesundheitsförderung und Prävention.

[26] Goodrich K, Kaambwa B, Al-Janabi H (2012). The inclusion of informal care in applied economic evaluation: a review. Value Health.

[27] Brouwer W, Rutten F, Koopmanschap M, Drummond M, McGuire A (2001). Costing in economic evaluations. Economic Evaluation in Health Care: Merging Theory with Practice.

[28] Posnett J, Jan S (1996). Indirect cost in economic evaluation: the opportunity cost of unpaid inputs. Health Econ.

[29] van den Berg B, Brouwer WBF, Koopmanschap MA (2004). Economic valuation of informal care. Eur J Health Econ.

[30] van den Berg B, Brouwer W, van Exel J, Koopmanschap M, van den Bos GAM, Rutten F (2006). Economic valuation of informal care: lessons from the application of the opportunity costs and proxy good methods. Soc Sci Med.

[31] van den Berg B, Ferrer-i-Carbonell A (2007). Monetary valuation of informal care: the well-being valuation method. Health Econ.

[32] Koopmanschap MA, van Exel JNA, van den Berg B, Brouwer WBF (2008). An overview of methods and applications to value informal care in economic evaluations of healthcare. Pharmacoeconomics.

[33] Weinstein MC, Siegel JE, Gold MR, Kamlet MS, Russell LB (1996). Recommendations of the panel on cost-effectiveness in health and medicine. JAMA.

[34] Brouwer WBF, Koopmanschap MA, Rutten FFH (1997). Productivity costs measurement through quality of life? a response to the recommendation of the Washington panel. Health Econ.

[35] Brouwer WBF, Koopmanschap MA, Rutten FFH (1997). Productivity costs in cost-effectiveness analysis: numerator or denominator: a further discussion. Health Econ.

[36] Sculpher M, Drummond M, McGuire A (2001). The role and estimation of productivity costs in economic evaluation. Economic Evaluation in Health Care: Merging Theory with Practice.

[37] Koopmanschap M, Burdorf A, Jacob K, Jan Meerding W, Brouwer W, Severens H (2005). Measuring productivity changes in economic evaluation: setting the research agenda. Pharmacoeconomics.

[38] Claxton K, Walker S, Palmer S, Sculpher M (2010). Appropriate Perspectives for Health Care Decisions (CHE Research Paper No. 54).

[39] Krol M, Brouwer W, Rutten F (2013). Productivity costs in economic evaluations: past, present, future. Pharmacoeconomics.

[40] Krol M, Brouwer W (2014). How to estimate productivity costs in economic evaluations. Pharmacoeconomics.

[41] Rappange DR, van Baal PHM, van Exel NJA, Feenstra TL, Rutten FFH, Brouwer WBF (2008). Unrelated medical costs in life-years gained: should they be included in economic evaluations of healthcare interventions?. Pharmacoeconomics.

[42] Meltzer D (1997). Accounting for future costs in medical cost-effectiveness analysis. J Health Econ.

[43] van Baal PHM, Feenstra TL, Hoogenveen RT, Ardine de Wit G, Brouwer WBF (2007). Unrelated medical care in life years gained and the cost utility of primary prevention: in search of a “perfect” cost–utility ratio. Health Econ.

[44] Felder S (2006). Lebenserwartung, medizinischer Fortschritt und Gesundheitsausgaben: Theorie und Empirie. Perspekt Wirtsch Suppl.

[45] Breyer F, Felder S (2006). Life expectancy and health care expenditures: a new calculation for Germany using the costs of dying. Health Policy.

[46] Gandjour A, Lauterbach KW (2005). Does prevention save costs?. J Health Econ.

[47] Gandjour A (2009). Aging diseases - do they prevent preventive health care from saving costs?. Health Econ.

[48] van Baal PHM, Feenstra TL, Polder JJ, Hoogenveen RT, Brouwer WBF (2011). Economic evaluation and the postponement of health care costs. Health Econ.

[49] Basu A, Meltzer D (2005). Implications of spillover effects within the family for medical cost-effectiveness analysis. J Health Econ.

[50] Cookson R, Drummond M, Weatherly H (2009). Explicit incorporation of equity considerations into economic evaluation of public health interventions. Health Econ Policy Law.

[51] Round R, Marshall B, Horton K (2005). Planning for Effective Health Promotion Evaluation.

[52] Rothgang H, Greß S, Niebuhr D, Wasem J (2004). Der Oregon Health Plan : ein Beispiel für “rationale Rationierung”?. Sozialer Fortschr Ger Rev Soc Policy.

[53] McGregor M (2003). Cost-utility analysis: Use QALYs only with great caution. Can Med Assoc J.

[54] Nord E, Daniels N, Kamlet M (2009). QALYs: some challenges. Value Health.

[55] Whitehead SJ, Ali S (2010). Health outcomes in economic evaluation: the QALY and utilities. Br Med Bull.

[56] Edlin R, Round J, McCabe C, Sculpher M, Claxton K, Cookson R (2008). Cost-Effectiveness Analysis and Ageism: A Review of the Theoretical Literature. A Report for the Department of Health.

[57] Salomon T, Rothgang H, AGil-Projekt (2011). Gesundheitsökonomische Evaluation bei Leistungen für Senioren: Führen diese zu einer Benachteiligung gesundheitsfördernder und präventiver Maßnahmen?. Prävent Gesundheitsförderung.

[58] Hickey DA, Barker M, McGee H, O’Boyle C (2005). Measuring health-related quality of life in older patient populations. Pharmacoeconomics.

[59] Cookson R, Culyer AJ. Measuring overall population health: the use and abuse of QALYs. In: Evidence-based Public Health: Effectiveness and Efficiency. Edited by Killoran A, Kelly MP, editors. Oxford: Oxford University Press; 2010:148–168.

[60] Weinstein MC (1988). A QALY Is a QALY Is a QALY - Or Is It?. J Health Econ.

[61] Makai P, Brouwer WBF, Koopmanschap MA, Stolk EA, Nieboer AP (2014). Quality of life instruments for economic evaluations in health and social care for older people: a systematic review. Soc Sci Med.

[62] Coast J, Flynn TN, Natarajan L, Sproston K, Lewis J, Louviere JJ, Peters TJ (2008). Valuing the ICECAP capability index for older people. Soc Sci Med.

[63] Grewal I, Lewis J, Flynn T, Brown J, Bond J, Coast J (2006). Developing attributes for a generic quality of life measure for older people: Preferences or capabilities?. Soc Sci Med.

[64] Couzner L, Ratcliffe J, Lester L, Flynn T, Crotty M (2013). Measuring and valuing quality of life for public health research: application of the ICECAP-O capability index in the Australian general population. Int J Public Health.

[65] Davis JC, Liu-Ambrose T, Richardson CG, Bryan S (2013). A comparison of the ICECAP-O with EQ-5D in a falls prevention clinical setting: are they complements or substitutes?. Qual Life Res.

[66] Makai P, Looman W, Adang E, Melis R, Stolk E, Fabbricotti I (2014). Cost-effectiveness of integrated care in frail elderly using the ICECAP-O and EQ-5D: does choice of instrument matter?. Eur J Health Econ.

[67] Makai P, Koopmanschap MA, Brouwer WB, Nieboer AA (2013). A validation of the ICECAP-O in a population of post-hospitalized older people in the Netherlands. Health Qual Life Outcomes.

[68] Makai P, Beckebans F, van Exel J, Brouwer WBF (2014). Quality of Life of Nursing Home Residents with Dementia: Validation of the German Version of the ICECAP-O. PLoS ONE..

[69] NICE (National Institute for Health and Care Excellence). Developing NICE Guidelines: The Manual. Process and methods guides; 2014.

